# P-1009. Invasive Fungal Disease is Rare in Multiple Myeloma Patients Treated with BCMA CAR-T Therapy

**DOI:** 10.1093/ofid/ofae631.1199

**Published:** 2025-01-29

**Authors:** Angelica Medina Pena, E Bridget Kim, Diana Cirstea, Sarah P Hammond, Jessica S Little

**Affiliations:** Brigham & Women's Hospital, Ocala, Florida; Massachusetts General Hospital, Boston, Massachusetts; Massachusetts General Hospital Cancer Center, Boston, Massachusetts; Massachusetts General Hospital, Boston, Massachusetts; Brigham and Women's Hospital, Boston, Massachusetts

## Abstract

**Background:**

B-cell maturation antigen (BCMA) chimeric antigen receptor T-cell (CAR-T) therapy has transformed treatment of relapsed/refractory multiple myeloma (MM). While this innovative therapy may introduce increased risks for infections, little is known about the characteristics and incidence of invasive fungal disease (IFD).

**Table 1.**
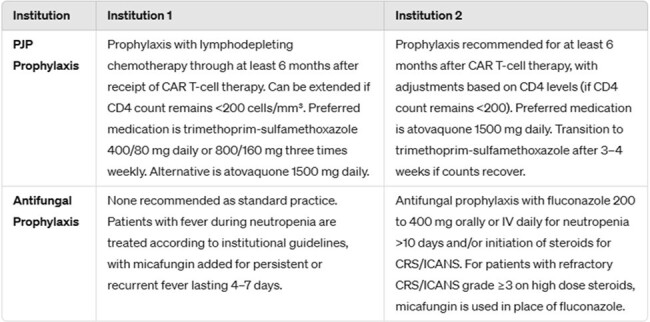
Institutional protocols for antifungal prophylaxis following immune effector cell therapy.

**Methods:**

We evaluated MM patients treated with first BCMA CAR-T therapy at 2 institutions November 2016 through October 2023. Table 1 summarizes institutional protocols for antifungal prophylaxis for immune effector cell (IEC) therapy. We documented IEC toxicities graded according to ASTCT and Lee Criteria. Proven or probable IFD was recorded from Day 0 through last follow-up, Day 365, or death according to the EORTC/MSGERC consensus criteria.

**Table 2.**
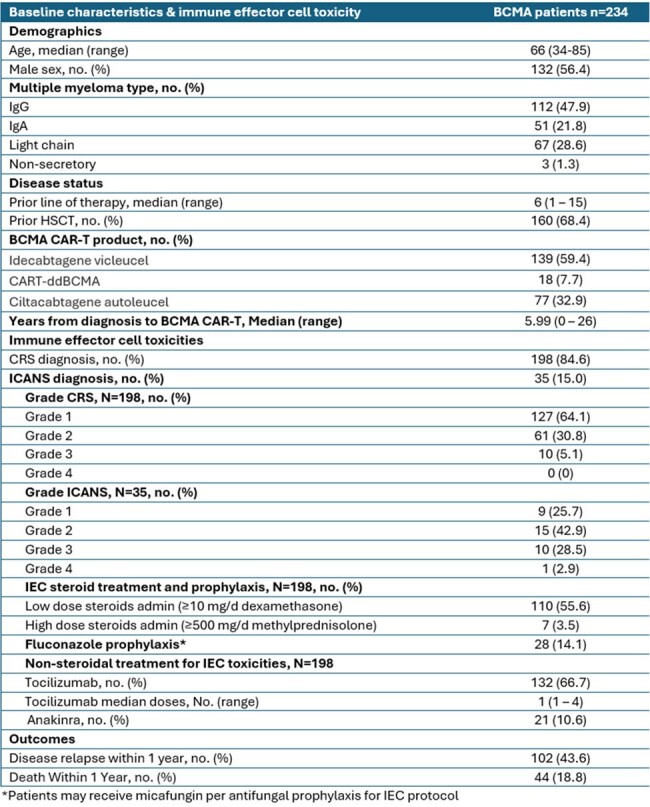
Baseline characteristics & immune effector cell toxicity

**Results:**

Among 234 patients in the cohort, median age was 66 years, and 56% were male. Patients had a median of 6 prior therapy lines, and 68% had undergone autologous stem cell transplant (Table 2). The most common IEC toxicity was cytokine release syndrome (CRS), observed in 84.6% of patients, primarily Grade 1-2 (Table 2). Four patients (1.7%) developed proven/probable IFD, including 1 with Candida albicans peritonitis and 3 with invasive aspergillosis. Among these 4 cases, the timing of diagnosis ranged from 6 to 104 days post-CAR-T infusion. All four had CRS/ICANS and received corticosteroids. Outcomes were mixed, with 3 cases reported as severe and 2 deaths within 30 days. One death occurred from Stenotrophomonas sepsis, and isolated cardiac Aspergillus was found incidentally on autopsy. On the second case, pulmonary Aspergillosis was found shortly before transitioning to comfort care due to hemophagocytic lymphohistiocytosis and *Staphylococcus aureus* pneumonia (Table 3).

**Table 3.**
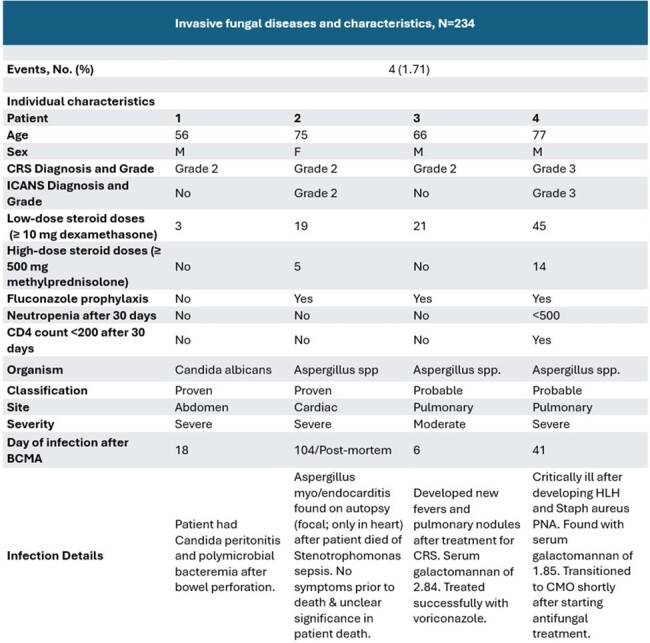
Characteristics of invasive fungal disease

**Conclusion:**

This is the largest study to date to provide details on the incidence and characteristics of IFD in BCMA CAR-T recipients. Here we demonstrate a low incidence of IFD despite a lack of routine antifungal prophylaxis. Nonetheless, these infections may be severe and appear to affect the most vulnerable with IEC toxicities with 50% of affected patients dying with active infection. Larger studies are needed to define underlying risk factors in this population.

**Disclosures:**

**Diana Cirstea, MD**, Sanofi: Advisor/Consultant **Sarah P. Hammond, MD**, Cidara: Grant/Research Support|F2G: Grant/Research Support|GSK: Grant/Research Support|Melinta: Advisor/Consultant|Pfizer: Advisor/Consultant|Roche: Advisor/Consultant|Scynexis: Grant/Research Support|Seres Therapeutics: Advisor/Consultant

